# Heavy oxygen recycled into the lithospheric mantle

**DOI:** 10.1038/s41598-019-45031-3

**Published:** 2019-06-19

**Authors:** Luigi Dallai, Gianluca Bianchini, Riccardo Avanzinelli, Claudio Natali, Sandro Conticelli

**Affiliations:** 1 Istituto di Geoscienze e Georisorse - Sede, CNR, Via G. Moruzzi, 1, I-56124 Pisa, Italy; 2 Dipartimento di Fisica e Scienze della Terra, Università degli Studi di Ferrara, Via G. Saragat, 1, I-44122 Ferrara, Italy; 3 Dipartimento di Scienze della Terra, Università degli Studi di Firenze, Via G. La Pira, 4, I-50121 Firenze, Italy; 4 Istituto di Geoscienze e Georisorse – Sede Secondaria di Firenze, CNR, Via G. La Pira, 4, I-50121 Firenze, Italy; 5 Istituto di Geologia Ambientale e Geoingegneria, CNR, Area della Ricerca Roma 1 - Montelibretti, Via Salaria km 29,300, I-00015 Monterotondo, Roma Italy

**Keywords:** Petrology, Geochemistry

## Abstract

Magmas in volcanic arcs have geochemical and isotopic signatures that can be related to mantle metasomatism due to fluids and melts released by the down-going oceanic crust and overlying sediments, which modify the chemistry and mineralogy of the mantle wedge. However, the effectiveness of subduction-related metasomatic processes is difficult to evaluate because the composition of arc magmas is often overprinted by interactions with crustal lithologies occurring during magma ascent and emplacement. Here, we show unequivocal evidence for recycling of continental crust components into the mantle. Veined peridotite xenoliths sampled from Tallante monogenetic volcanoes in the Betic Cordillera (southern Spain) provide insights for mantle domains that reacted with Si-rich melts derived by partial melting of subducted crustal material. Felsic veins crosscutting peridotite and the surrounding orthopyroxene-rich metasomatic aureoles show the highest ^18^O/^16^O ratios measured to date in upper mantle assemblages worldwide. The anomalously high oxygen isotope compositions, coupled with very high ^87^Sr/^86^Sr values, imply the continental crust origin of the injected melts. Isotopic anomalies are progressively attenuated in peridotite away from the veins, showing ^18^O isotope variations well correlated with the amount of newly formed orthopyroxene. Diffusion may also affect the isotope ratios of mantle rocks undergoing crustal metasomatism due to the relaxation of ^18^O isotope anomalies to normal mantle values through time. Overall, the data define an O isotope “benchmark” allowing discrimination between mantle sources that attained re-equilibration after metasomatism (>5 Myr) and those affected by more recent subduction-derived enrichment processes.

## Introduction

Mantle wedges at destructive plate margins are significantly heterogeneous in chemical and isotopic compositions due to the reaction of the original mantle mineralogy with fluids and/or melts from the subducted slab^1–7^. Dehydration and/or melting of material from the subducted slab produce H_2_O- and CO_2_-rich metasomatizing agents, which in turn may trigger partial melting in the asthenospheric mantle wedge responsible for arc magmatism^8–11^. Sub-continental lithospheric mantle interacts similarly with percolating fluids or melts released by the subducting lithosphere^12–18^.

Evidence of these processes is found in the chemical and isotopic compositions of subduction-derived magmatic rocks worldwide^8,13,19^. In several cases, however, especially in continental settings, shallow-level crustal contamination can also modify the composition of the erupted magmas, making it difficult to discriminate between processes occurring within the mantle source and those affecting the magmas en route to the surface^17,18,20^.

Oxygen isotope investigations provide evidence for recycling within the mantle of fluids/melt derived from the subducted oceanic crust^21–23^. The limited oxygen isotope fractionation at mantle temperatures and the narrow O isotope compositional variations in “typical” mantle rocks (δ^18^O_ol_ = 5.18 ± 0.28‰; δ^18^O_opx_ = 5.69 ± 0.28‰; δ^18^O_cpx_ = 5.57 ± 0.36‰)^24^ make oxygen isotopes a powerful tool for identifying recycled crustal material in the mantle^23,25^. It is difficult, however, to define the specific (oceanic *vs*. continental) nature of the subducted components, and evidence for recycling of continental crust is generally rare and elusive^26^. Moreover, as highlighted above, high δ^18^O values recorded in erupted subduction-related lavas can also (and often) be due to crustal contamination, and it is frequently difficult to unequivocally discriminate between the two processes^27^.

In this study, we determined the O isotope compositions, coupled with new Sr and Nd isotope data, on primary minerals from peridotite mantle xenoliths and felsic veins in composite mantle xenoliths from Tallante monogenetic volcanoes, Betic Cordillera, southern Spain^28–32^. The host lavas were erupted in a post-collisional tectonic setting (cf. Supplementary Paragraph 1), exhuming abundant ultramafic mantle xenoliths (cf. Supplementary Paragraphs 2 and 3), which likely represent portions of a supra-subduction mantle wedge^28–34^. Therefore, the studied samples provide an almost unique opportunity to directly study the effect of mantle metasomatism without any possible influence of shallow-level crustal contamination.

## Extreme Oxygen Isotope Variability in Mantle Minerals from Tallante

**Figure 1:**
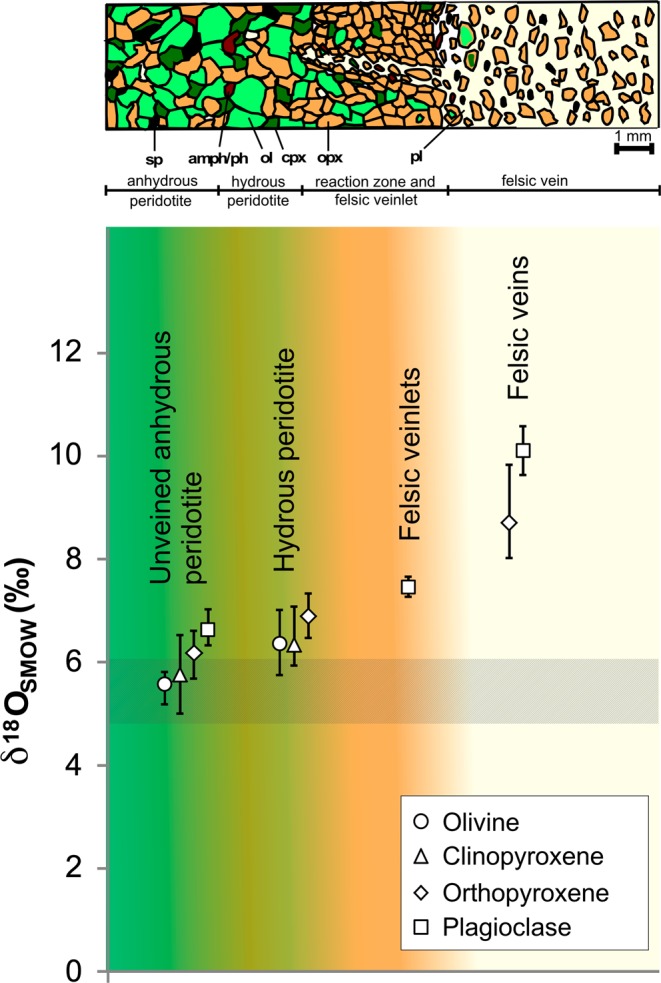
(**a**) Petrographic sketch of lithological and petrographic variations observed in composite mantle xenoliths from Tallante (southern Spain); peridotite is crosscut by felsic veins. (**b**) Oxygen isotope variations in minerals from Tallante composite mantle xenoliths; average and maximum/minimum values observed in the studied xenoliths are expressed by symbols and bars, respectively, whereas shaded areas represent the notional isotope values expected in uncontaminated mantle^24^.

The peridotite mantle xenoliths from Tallante display proto-granular textures and a mineralogy composed of orthopyroxene (opx), olivine (ol), clinopyroxene (cpx), spinel (sp) and occasional plagioclase (pl). In some composite xenoliths, centimetre- to millimetre-sized felsic veins crosscut the peridotite (Fig. 1). These veins mainly consist of plagioclase and orthopyroxene with accessory amphibole (amph), phlogopite, quartz, apatite, monazite/huttonite, rutile, and zircon (see also Supplementary Paragraph 2). *In situ* U-Pb dating of paragenetic zircons yields ages between 2.2 and 6.8 Ma^35^, suggesting a close link to the Tertiary subduction process, which ultimately led to the formation of the Betic Cordillera.

To schematise, the studied xenoliths are divided into three broad groups representing different portions of the lithospheric mantle under the Betic region: (i) anhydrous peridotite xenoliths, sometimes containing plagioclase; (ii) orthopyroxene-rich, amphibole- and plagioclase-bearing xenoliths; and (iii) composite xenoliths with felsic veins showing clear evidence of reaction with percolating silica-rich melts. Group ii is interpreted as transitional between the unveined and veined mantle domains represented by group i and group iii, respectively.

Oxygen isotope compositions (δ^18^O = [R_sample_/R_ref_ − 1] * 1000; R = ^18^O/^16^O) were measured on distinct mineral phases of the three groups of Tallante xenoliths delineated above (Supplementary Paragraph 4 and Supplementary Table 1). The largest variability of δ^18^O values is shown in the composite xenoliths (group iii) crosscut by felsic veins (Fig. 1a). The minerals forming the veins preserve the highest δ^18^O values (sample TL112: δ^18^O_opx_ + 9.84‰, δ^18^O_pl_ + 10.56‰; sample TL5: δ^18^O_pl_ + 9.63‰, δ^18^O_opx_ + 8.02‰). These δ^18^O values are among the highest ever recorded in mantle phases (see Supplementary Material). Orthopyroxene from the vein/peridotite reaction domains still shows anomalously high O isotope compositions (δ^18^O_opx_ = 7.32–8.38‰), whereas the anomalies are less pronounced in the surrounding peridotite matrix. Plagioclase within smaller felsic veinlets shows lower δ^18^O values (sample TL117: δ^18^O_pl_ = 7.33‰; sample TL347: δ^18^O_pl_ = 7.58‰) than that from thicker veins, yet these values are still higher than those of the surrounding peridotite domains, suggesting that the larger the vein is, the higher the δ^18^O values of orthopyroxene and plagioclase contained therein.

The isotopic anomalies persist, although to a lesser extent, in unveined anhydrous peridotite xenoliths (group i) that range in composition from harzburgites to lherzolites (Supplementary Paragraph 4), especially in samples containing plagioclase. The δ^18^O values in minerals from these samples are still greater than typical mantle values (5.19‰ ≤ δ^18^O_ol_ ≤ 5.78‰; 5.68‰ ≤ δ^18^O_opx_ ≤ 6.47‰; 5.03‰ ≤ δ^18^O_cpx_ ≤ 6.62‰) and similar to those measured in O-enriched sub-arc mantle peridotite^21,22,25^, showing large δ^18^O variabilities within single mineral phases, which correlate well with the modal contents of the constituent phases (Supplementary Paragraph 5).

Overall, our samples define a broad picture where δ^18^O decreases away from the metasomatic veins through the reaction zones and in the surrounding peridotite (Fig. 1a,b); within this framework, the orthopyroxene-rich, amphibole- and plagioclase-bearing harzburgite xenolith (TL23) coherently represents a distal portion of the reaction zone triggered by felsic veining, having δ^18^O values (δ^18^O_ol_ = 6.99‰, δ^18^O_opx_ = 7.33‰, δ^18^O_cpx_ = 7.02‰) similar to those of the orthopyroxene found in the reaction zone of the veined xenoliths.

## Nature and Origin of the Percolating Melts Producing the Veins

The nature of orthopyroxene-rich mantle domains is widely debated^4,36–39^. Partial melts of subducted material injected into pre-existing supra-subduction mantle domains have been found to produce secondary orthopyroxene when SiO_2_-rich percolating melts react with peridotite^9,40^.

The occurrence of rare quartz crystals within orthopyroxene-plagioclase veinlets of composite mantle xenoliths further constrains the silica-oversaturated nature of the percolating melts and their plausible derivation from partial melting of subducted material^41^. New ^87^Sr/^86^Sr and ^143^Nd/^144^Nd values (Supplementary Paragraph 4 and Supplementary Table 2) obtained in plagioclase and clinopyroxene crystals from the veins of the composite xenoliths provide further clear evidence (together with the extreme δ^18^O values) for a crustal, subduction-related origin of the metasomatizing agent. Indeed, plagioclase crystals from the thicker felsic vein are characterised by highly radiogenic Sr (^87^Sr/^86^Sr = 0.71266) and non-radiogenic Nd isotope compositions (^143^Nd/^144^Nd = 0.51260) that can be attained only if the percolating melt originated from subducted continental crust material. This material could have been recycled in the subcontinental mantle through slab delamination processes occurring during the Betic continental collision and may be represented by meta-sedimentary xenoliths exhumed in the same volcanic centre as the studied mantle xenoliths^35^. These metasedimentary xenoliths, which equilibrated under P-T conditions similar to those of the co-existing mantle xenoliths (0.7 GPa, 1050 °C), have silica/alumina-rich bulk compositions and consist mainly of quartz, plagioclase, garnet, spinel, and ilmenite ± orthopyroxene ± sillimanite ± graphite.

Plagioclase and clinopyroxene from smaller (millimetre-sized) veinlets are characterised by lower radiogenic Sr (^87^Sr/^86^Sr = 0.70286–0.70409) and higher Nd (^143^Nd/^144^Nd = 0.51282–0.51318), which are similar to the values in clinopyroxene from the surrounding peridotite matrix. The observed variability in Sr and Nd isotopes (Supplementary Table 2) suggests that vein and veinlet formation occurred through metasomatic reactions between the primary mantle minerals and melts of variable composition and involved different melt/wall rock proportions. Due to an extreme chemical potential gradient, percolating metasomatic melts are highly reactive; thus, their initial compositions are difficult to assess. Any melt would interact extensively with the peridotite of the mantle wedge, given its alkali-rich and silica-rich nature^42,43^, and would equilibrate with mantle minerals^44^. Orthopyroxene oversaturation is achieved during melt evolution after olivine is completely consumed by the melt-peridotite reaction^9,36,45,46^. In this scenario, the minerals in the felsic veins preserve δ^18^O values that are the closest to those of the percolating felsic melts, whereas in the adjacent domains, progressively lower δ^18^O values attest less melt/matrix interaction.

## Oxygen Isotope Compositions of Mantle Domains Surrounding the Felsic Veins

Silica-rich melts released by the partial melting of subducted continental crust components appear to be channelled into lithospheric fractures through the pre-existing peridotite, inducing sandwich texture with centimetre- (TL112) to millimetre-sized (TL117, TL347) veins bordered by newly formed metasomatic orthopyroxene + plagioclase domains.

Orthopyroxene and plagioclase in the reaction zone are produced by metasomatism according to the following reactions^36,44,47^:

1 $${\rm{ol}}+{{\rm{SiO}}}_{2} \mbox{-} {\rm{rich}}\,{{\rm{melt}}}_{1}={\rm{opx}}+{{\rm{melt}}}_{2}$$

and

2 $${\rm{cpx}}+{\rm{sp}}+{{\rm{SiO}}}_{2}-{\rm{rich}}\,{{\rm{melt}}}_{1}={\rm{pl}}+{\rm{opx}}+{\rm{quartz}}\pm {{\rm{melt}}}_{2}$$

Orthopyroxene from the boundary surrounding the vein still shows anomalous O isotope compositions, which are significantly lower than those of the mineral phases within the vein. This contrast is consistent with the metasomatic reactions described above, where most of the oxygen in the newly formed orthopyroxene derives from the unmodified peridotite, and only a subordinate amount is instead inherited from the percolating melt.

**Figure 2:**
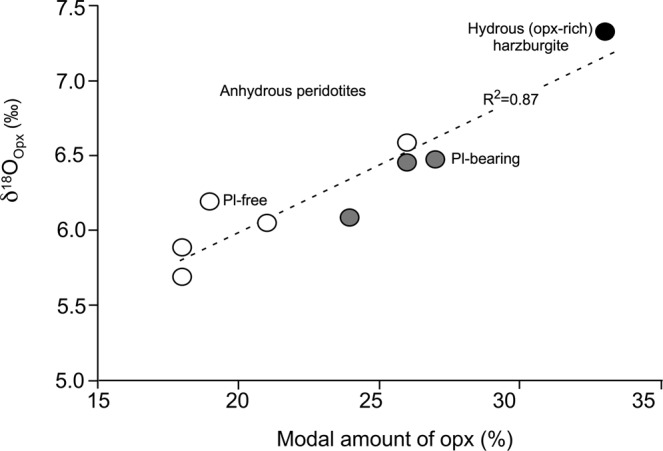
Relationships between modal orthopyroxene amount and the relative oxygen isotope composition in unveined peridotite mantle xenoliths from Tallante (southern Spain). Anhydrous xenoliths (group i) are subdivided into plagioclase-free (open circles) and plagioclase-bearing (grey filled circles), whereas orthopyroxene-rich, amphibole- and plagioclase-bearing xenoliths (group ii) are recorded as black circles.

In addition, a positive covariation trend between modal contents of orthopyroxene and δ^18^O_opx_ is observed in the vein-free ultramafic xenoliths (groups i and ii; Fig. 2), which is accompanied by concomitant crystallisation of plagioclase and approximately positive covariation trends between orthopyroxene/clinopyroxene and orthopyroxene/olivine values *vs*. δ^18^O_opx_ (Supplementary Fig. 9a,b, respectively). This correlation plausibly indicates formation of new orthopyroxene during the silica-rich melt-peridotite reaction, even in some unveined anhydrous peridotite domains (grey field circles in Fig. 2 and Supplementary Fig. 9a,b).

The occurrence of samples with mineral phases showing δ^18^O values variably higher than those of the unmodified peridotitic mantle, on one hand, and of samples with mineral phases showing typical crustal δ^18^O values, on the other hand, suggests that the silica-rich metasomatic melt was progressively consumed and that it evolved during reaction with wall rock peridotite.

**Figure 3:**
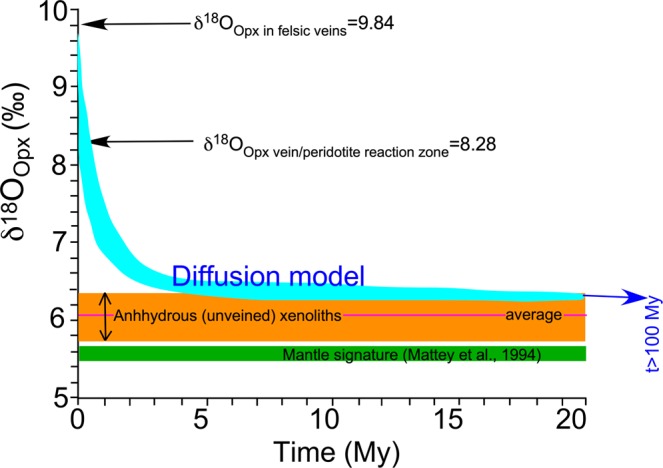
Oxygen isotope diffusion model calculating the time necessary to reset the isotopic heterogeneities induced by the ^18^O-rich veining metasomatic agents. The blue ribbon delineates the expected temporal variation of the oxygen isotopic composition, assuming the diffusion parameters proposed by Ingrin *et al*.^51^ for starting δ^18^O_opx_ values of 9.84 and 8.28‰. See Supplementary Material and Supplementary Table 3 for further details.

The metasomatic orthopyroxene-rich domains enveloping the veins subsequently equilibrated with the surrounding peridotite, acting as an armour against exotic material. Accordingly, the metasomatic reaction may be explained as an initial reactive porous flow reaction, followed by diffusion-assisted re-equilibration. Indeed, further oxygen isotope heterogeneities are likely derived from incomplete O isotope equilibration towards mantle-like values due to limited intra-crystalline diffusion, which is affected by the age of exotic melt injection into the peridotite, the initial oxygen isotope composition of the contaminant, the occurrence of fluid phases, and the chemical-physical characteristics of the mineral assemblage forming the peridotite wall rock. We can use oxygen isotope diffusion data to calculate the temporal variation in δ^18^O and the time required for complete re-equilibration. Note that the time required to physically digest exotic crustal material under mantle conditions clearly differs from that required to partly or completely obliterate the oxygen isotope signature. Oxygen diffusion at mantle temperatures is thought to be rapid enough to erase disequilibrium among minerals on a million-year time scale^48,49^. It follows that if the δ^18^O values of orthopyroxene in the veins represent the initial values of the mineral phase newly crystallised in the mantle, we can calculate the compositional variations due to O isotope diffusive re-equilibration with the surrounding mantle. Using the empirical oxygen isotope geothermometer of Matthews *et al*.^50^, the temperature of equilibration based on the Δ^18^O_pl-opx_ is estimated at 930 °C. This result is consistent with those of previous studies^31^, as the temperature of equilibration in the felsic veins estimated based on the compositions of the plagioclase-amphibole pair constrains the temperature of veining to above 850 °C, whereas the equilibration temperature of the peridotite domains (based on the compositions of the orthopyroxene-clinopyroxene pair) is in the range between 830 and 1030 °C. At these temperatures, model calculations predict a rapid decrease in δ^18^O_opx_ values over the first 5–6 Myr (Fig. 3 and Supplementary Fig. 3). After this timespan, the orthopyroxene O isotope composition shows small variation with time, and the average δ^18^O value remains slightly higher than 6‰. These values are similar to the values measured in some anhydrous unveined peridotite mantle xenoliths (open circles in Fig. 2 and Supplementary Fig. 9), indicating that the local sub-continental lithospheric mantle includes portions completely re-equilibrated after earlier metasomatic events. On the other hand, other portions of such mantle, represented by the veined and the orthopyroxene-rich, amphibole- and plagioclase-bearing xenoliths, still preserve isotopic disequilibrium, thus implying that metasomatism continued up until shortly before the xenolith were entrained and exhumed, in agreement with the available U-Pb zircon dating^35^. Upon melting, such a mantle could have generated magmas with high δ^18^O, even before any possible modification due to shallow-level crustal contamination during magma ascent.

## Implications for Magma Generation in the Betic Post-Collisional Setting

**Figure 4:**
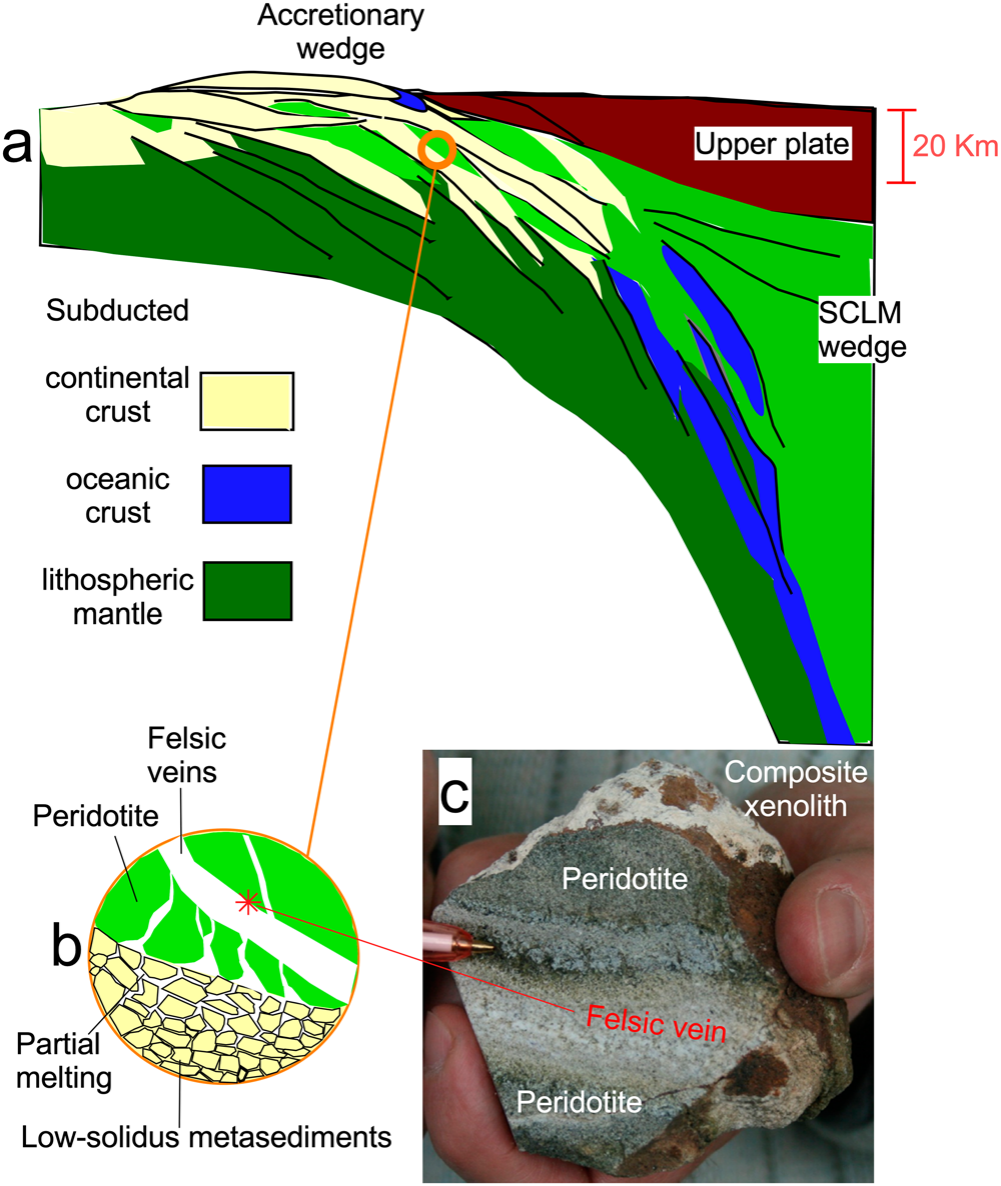
(**a**) Cartoon showing a cross-sectional sketch of crustal recycling during continental collision, after which oceanic lithosphere is completely subducted. Note that the model invokes crust-mantle interlayering (melanges). Partial melting of the crustal domains generates felsic melts that percolate through the peridotite domains and react with them. (**b**) The crustal melts react with the surrounding peridotite and freeze to form the metasomatic veins, recycling heavy oxygen into the mantle. (**c**) Such a process is recorded in the composite mantle xenoliths (e.g., TL112) erupted at Tallante.

The complex processes revealed by our data are synthesised in the cartoon of Fig. 4, which illustrates the possible recycling of continental crust slivers during late-stage subduction processes of the continental collision in the Betic area. The process generates inter-layering of mantle and (low solidus) crustal lithotypes, i.e., the formation of crust-mantle melanges that are sometimes observed in orogenic peridotite massifs (Supplementary Paragraph 3). The genesis of magmas in post-collisional settings potentially involves extremely heterogeneous sources, in which mantle domains have been variously hybridised by metasomatism with crustal melts. This factor implies that exotic post-collisional magmas can inherit ^18^O-rich isotope compositions directly from metasomatised mantle sources and that their isotope signature depends on the time interval between metasomatism and partial melting of the mantle source and eventually on crustal contamination during magma ascent to the surface. According to this hypothesis, δ^18^O_opx_ values of 6.2‰ would be the maximum that can be expected in the Betic magmas produced by partial melting of equilibrated mantle domains (where the time lapse between metasomatism and magma genesis exceeds 5 Myr). Higher δ^18^O values necessarily imply nearly contemporaneous source veining/metasomatism and magma genesis or have to be attributed to crustal contamination during magma ascent.

## Supplementary information

Supplementary Material and Supplementary Data
