# Supplementary material for: Heavy oxygen recycled into the lithospheric mantle
Source: Sci Rep. 2019 Jun 19;9:8793. doi: 10.1038/s41598-019-45031-3 (PMC6584624; doi:10.1038/s41598-019-45031-3)
Supplement: Supplementary file 1 — Supplementary Material and Supplementary Data [file 41598_2019_45031_MOESM1_ESM.pdf]

SCIENTIFIC REPORTS

Supplementary data and information to

Heavy oxygen recycled into the lithospheric mantle

Luigi Dallai[1], Gianluca Bianchini[2], Riccardo Avanzinelli[3,4], Claudio Natali[2,3] & Sandro Conticelli[3,4,5]

[1] Istituto di Geoscienze e Georisorse - Sede, CNR, Via G. Moruzzi, 1, I-56124, Pisa, Italy

[2] Dipartimento di Fisica e Scienze della Terra, Università degli Studi di Ferrara, Via G. Saragat, 1, I-44122, Ferrara, Italy

[3] Dipartimento di Scienze della Terra, Università degli Studi di Firenze, Via G. La Pira, 4, I-50121, Firenze, Italy

[4] Istituto di Geoscienze e Georisorse – Sede Secondaria di Firenze, CNR, Via G. La Pira, 4, I-50121, Firenze, Italy

[5] Istituto di Geologia Ambientale e Geoingegneria, CNR, Area della Ricerca Roma1 - Montelibretti, Via Salaria km 29,300, I-00015, Monterotondo (Roma), Italy

1. Geological outlines

1.1. Geodynamic setting

The Gibraltar arc in the westernmost Mediterranean includes the Betic and Rif orogens, which surround the Alborán Sea basin and reflect a complex evolution of subduction, slab roll back and fragmentation that were induced by continental plate convergence. In particular, the Betic Cordillera in southern Spain is constituted by an "External" thrust belt that exposes Triassic to Cenozoic sediments, and a metamorphic domain known as the "Internal" Zones. The latter is formed by a series of Variscan crystalline units and Mesozoic sedimentary sequences. The orogen resulted from the Tertiary closure of the western Tethys Ocean and subsequent continental collisions that involved the Africa and Eurasia (including Iberia) plates (Faccenna et al., 2004; Puga et al., 2011; Platt et al., 2013). Most authors currently believe to the existence of a subducted slab under the area, as recorded by tomographic images and deep seismicity distribution (Wortel & Spakman, 2000; Faccenna et al., 2004; Platt et al., 2013 and references therein).

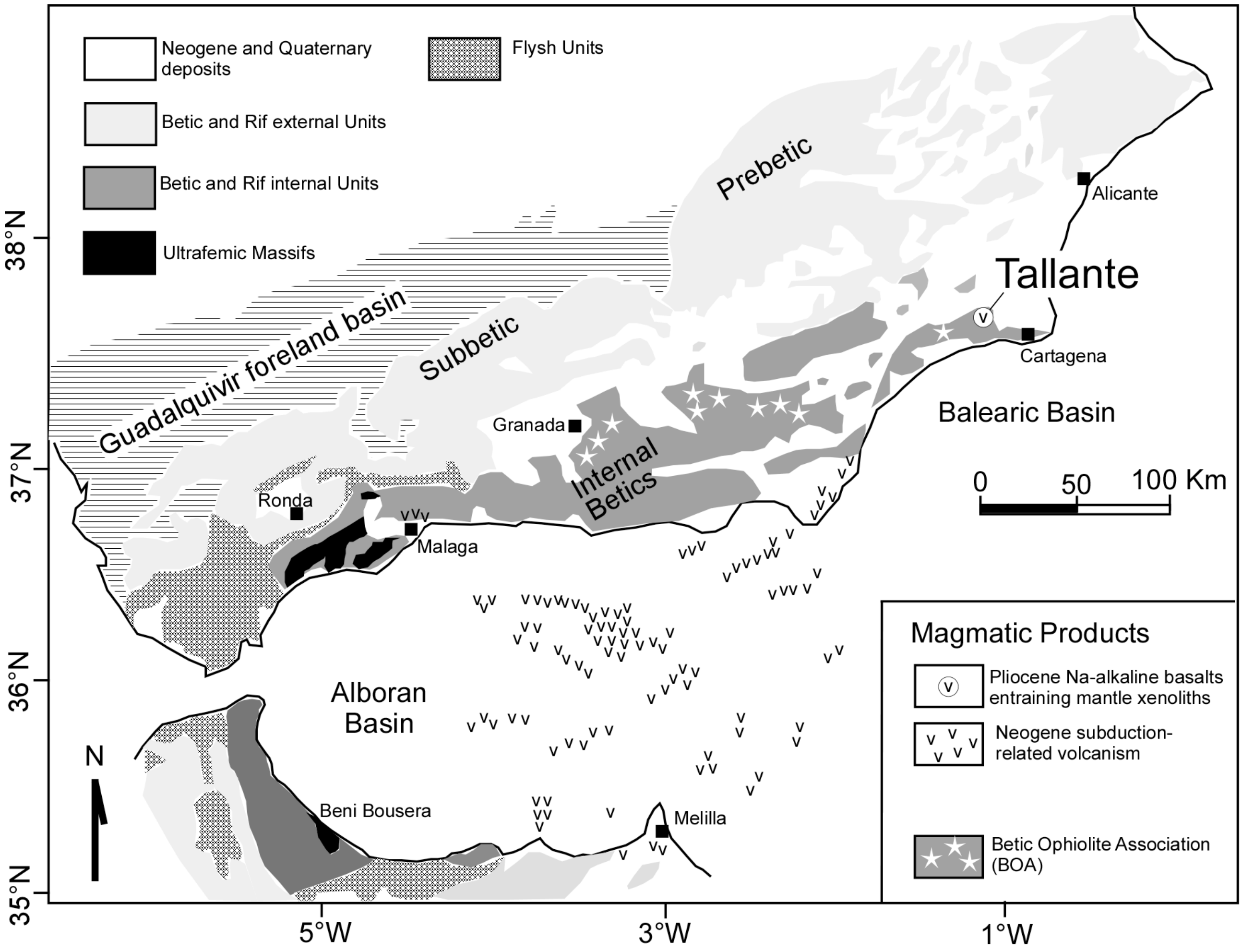

Supplementary Fig. 1. Simplified geological sketch map of the circum-Alborán area (redrawn after Bianchini et al., 2011). Note that the Gibraltar Arc has two arms represented by the Betic Cordillera of southern Spain and the Rif chain of northern Morocco, which land-locked the Alboran Basin situated in the internal part of the Arc. In particular, the Betic Cordillera of southern Spain is divided in external and internal units.

1.2. Volcanism

The South East Volcanic Province of Spain (SEVP) is an example of orogenic (i.e. subduction related) and post-orogenic magmatism developed during the opening of the Alborán back-arck basin (Duggen et al., 2005; Mattei et al., 2014).

The magmatism was characterised by distinct events that show a peculiar distribution in time and space: calc-alkaline products outcrop at Sierra de Gata (12-8 Ma), High-K calcalkaline (and shoshonitic) rocks at Mazárron and ultrapotassic rocks at Murcia (10-6 Ma)(e.g., Benito et al., 1999; Turner et al, 1999; Duggen et al., 2005, 2008; Conticelli et al., 2009; Mattei et al., 2014).

After a pause of four million years (e.g., Ancochea & Nixon, 1987; Cebria et al., 2009), volcanic activity renewed in the locality of Tallante and surroundings with the eruption of within-plate Na-alkaline basalts, to form a monogenetic volcanoes field, that entrained and exhumed deep seated xenoliths.

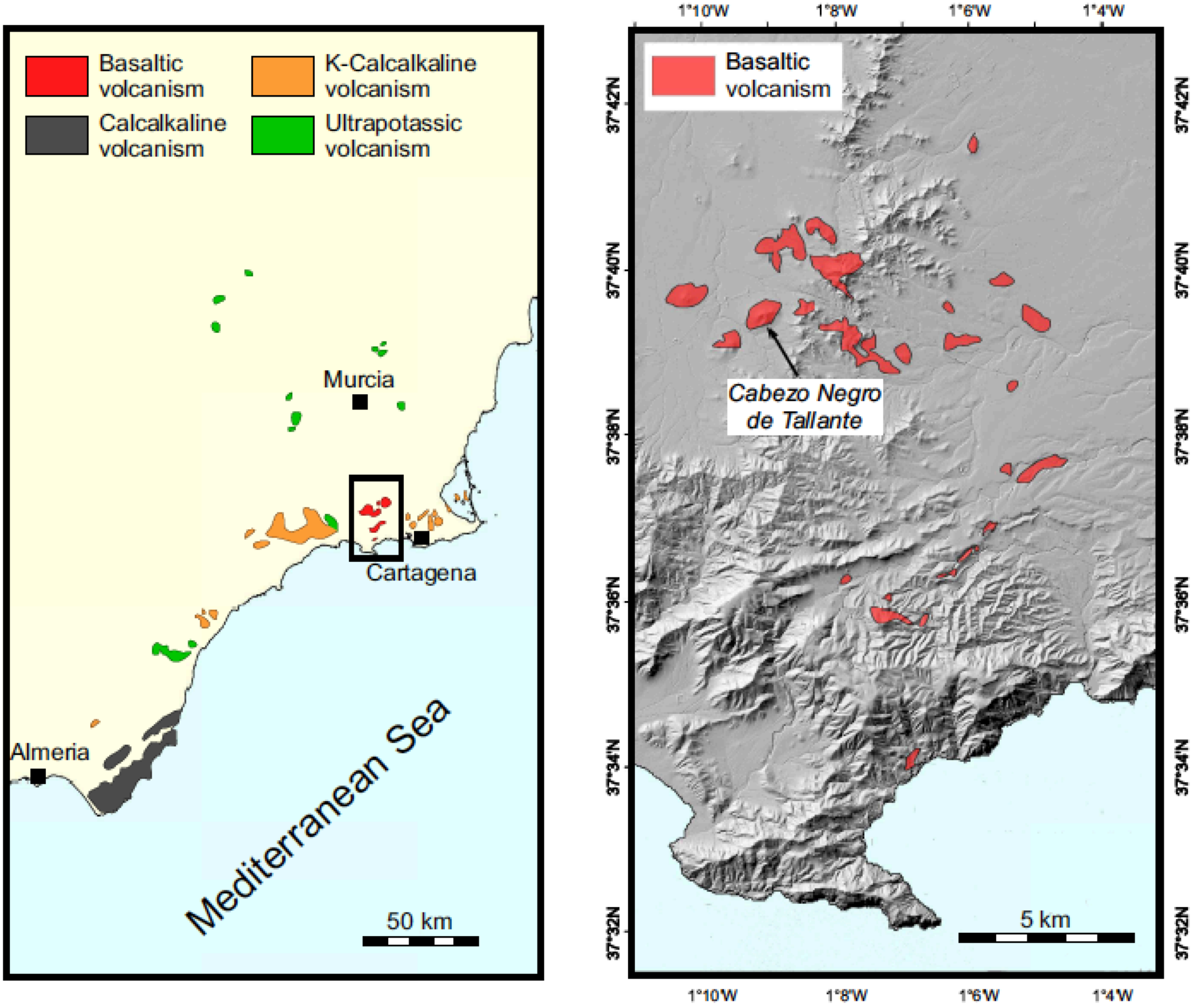

Supplementary Fig. 2. Geographic distribution of the South East Volcanic Province of Spain (SEVP) including the volcanic field of of Tallante (redrawn after Cebriá et al., 2009).

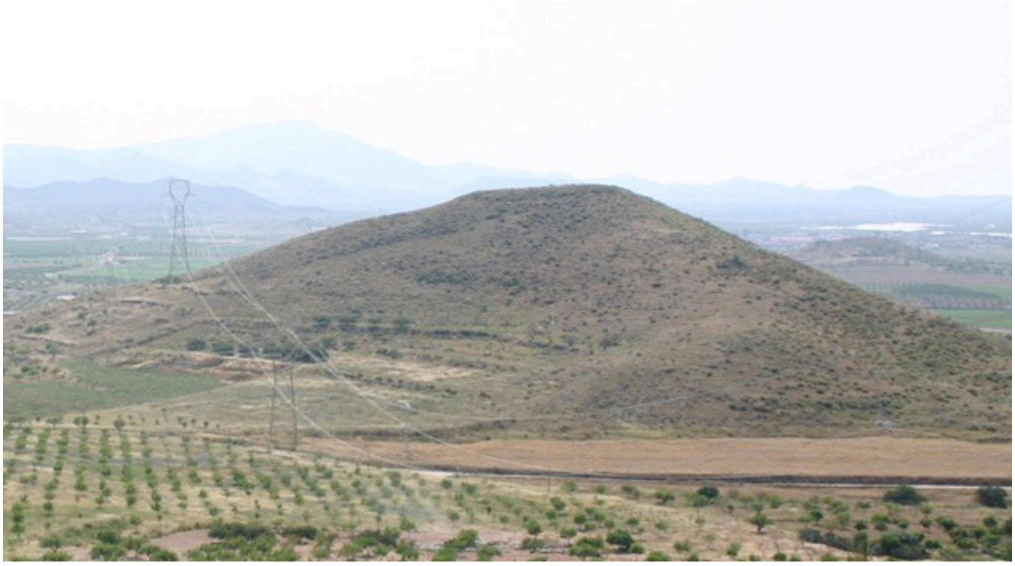

Supplementary Fig. 3. The volcanic centre of Cabezo Negro de Tallante, where the studied xenoliths have been collected (Photograph by Gianluca Bianchini).

2. The deep seated xenoliths from Tallante

2.1. The mantle xenoliths from Tallante: an overview

The xenoliths from the volcanic centre of Cabezo Negro de Tallante display notable size (up to 20 cm) and freshness and attracted an intense petrological interest reflected in a large number of studies (Dupuy et al., 1986; Ancochea and Nixon, 1987; Capedri et al., 1989; Turner et al., 1999; Kogarko et al., 2001; Arai et al., 2003; Beccaluva et al., 2004; Shimizu et al., 2004; 2008; Coltorti et al., 2007; Rampone et al., 2010; Bianchini et al., 2011; Martelli et al., 2011; Konc et al., 2012; Hidas et al., 2016; Marchesi et al., 2017).

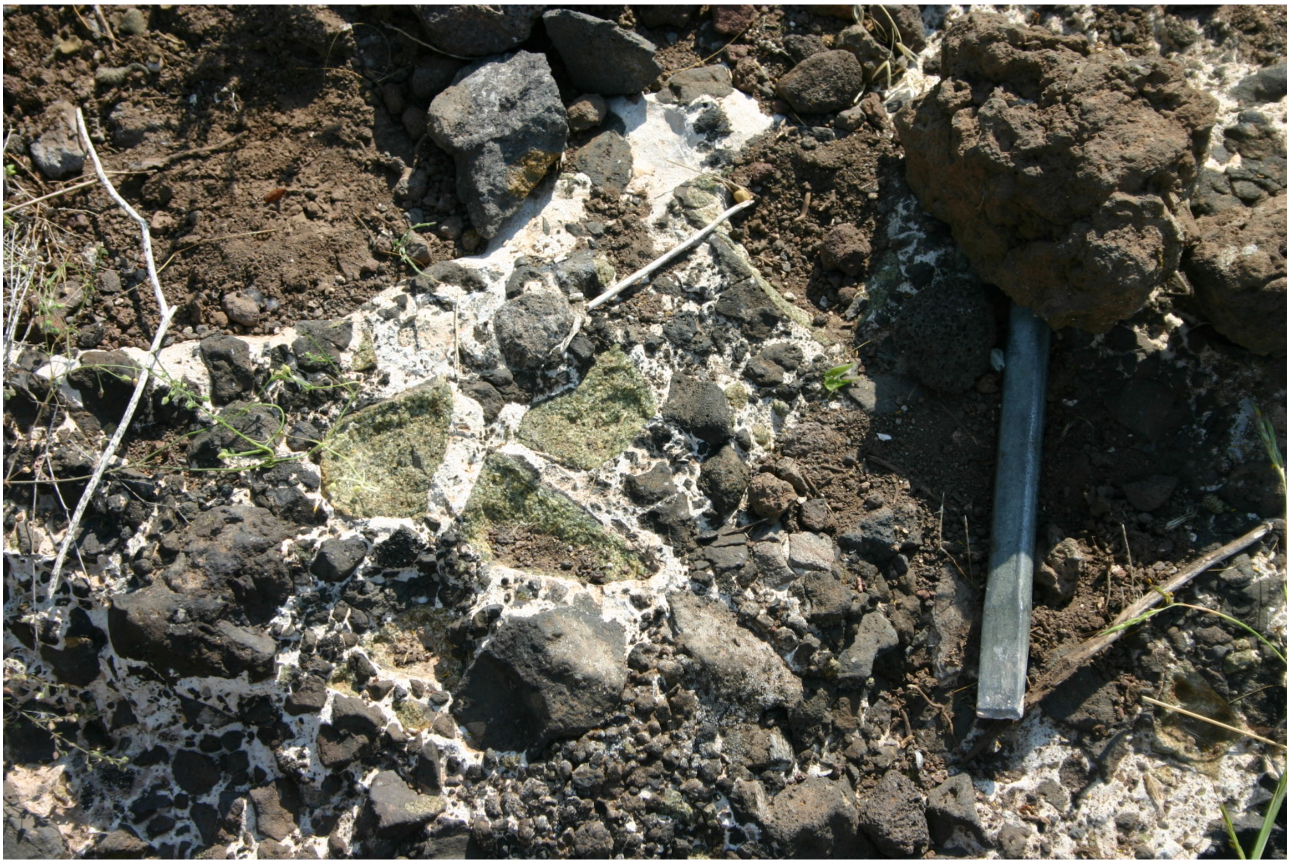

Supplementary Fig. 4. Photography showing a volcanic breccia containing abundant mantle xenoliths at the volcanic centre of Cabezo Negro de Tallante (Photograph by Gianluca Bianchini).

Most of the mantle xenoliths are represented by (anhydrous) spinel-plagioclase protogranular peridotites equilibrated at P-T conditions of 830–1000 °C and 0.7–0.9 GPa (Bianchini et al., 2011), but composite xenoliths formed by peridotite crosscut by igneous veins are also observed.

The mentioned igneous veins can, in turn be subdivided in mafic (dark) and felsic (whitish) veins. Mafic veins are mainly composed by clinopyroxene ± amphibole ± phlogopite and subordinate plagioclase and have silica-undersaturated bulk composition resembling that of the host alkaline basalts. Felsic veins, on the other hand, are mainly composed by plagioclase ±

orthopyroxene ± aluminiferous spinel (Dupuy et al., 1986; Ancochea and Nixon, 1987; Capedri et al., 1989; Beccaluva et al., 2004; Bianchini et al., 2011) and are clearly related to silica-oversaturated melts, as testified by the accessory presence of quartz (*cfr.* Arai et al., 2003; Shimizu et al., 2004). These felsic veins, having thickness variable from sub-millimetric to few centimetres, are always armoured by an orthopyroxene-rich reaction zone, which indicates that the upraising silica-rich melt reacted with the host–peridotite.

Noteworthy, the presence of composite mantle peridotite xenoliths crosscut by such felsic veins is, as far as we know, a peculiarity observed only in the Tallante xenoliths suite, which therefore represents an extraordinary study-case.

Accessory minerals, such as amphibole (pargasite) and phlogopite, but also graphite, apatite, monazite, huttonite/thorite, zircon, rutile, become more abundant in the thinner veinlets that plausibly represent apophyses of larger veins (and in the adjoining reaction zones), thus suggesting that incompatible elements tend to concentrate in the final stages of veining and metasomatism of the surrounding peridotite mantle.

2.2. Petrographic features of the studied mantle xenoliths

This study is based on 13 mantle xenoliths from Tallante that can be subdivided in three groups: i) anhydrous peridotite xenoliths, ii) hydrous orthopyroxene-rich, amphibole-bearing xenoliths and iii) composite xenoliths with felsic veins showing clear evidence of reaction with percolating silica-rich melts.

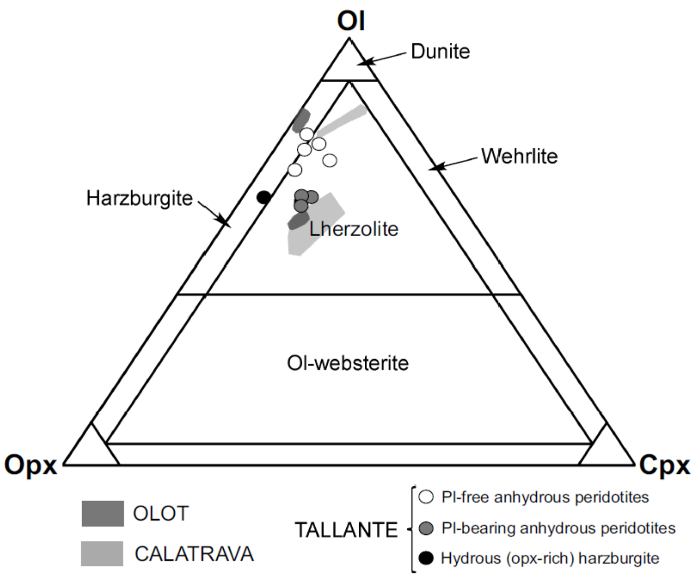

Supplementary Fig. 5. Modal composition (data from Beccaluva et al., 2004) of the unveined Tallante xenoliths taken into consideration in this study, compared with the composition of xenoliths from other Spanish volcanic occurrences (data from Bianchini et al., 2007; 2010).

Unveined anhydrous spinel-peridotite xenoliths (i) are characterised by protogranular texture and can in turn be subdivided in harzburgites (TL1, TL24), and lherzolites (TL16, TL22, TL14, TL20, TL45, TL53) sometimes containing enough modal plagioclase to be separated by hand-picking (TL14, TL20, TL22, TL45). The unveined orthopyroxene-rich amphibole bearing harzburgite (ii) also contains enough modal plagioclase to be separated by hand-picking (TL23).

In composite xenoliths (iii) protogranular peridotite portions are crosscut by felsic veins of variable size; among these samples, TL112 has a centimetric vein mainly composed of plagioclase and orthopyroxene (sometimes forming symplectites) with minor quartz, phlogopite, graphite; TL5 has a felsic vein made of plagioclase and orthopyroxene (sometimes forming symplectites) with accessory amphibole and phlogopite; other composite xenoliths are represented by sample TL117 and TL347 in which peridotite is crosscut by millimetric felsic veinlets composed by orthopyroxene and plagioclase with accessory amphibole and phlogopite and traces of apatite, monazite, zircon, thorite/huttonite and rutile.

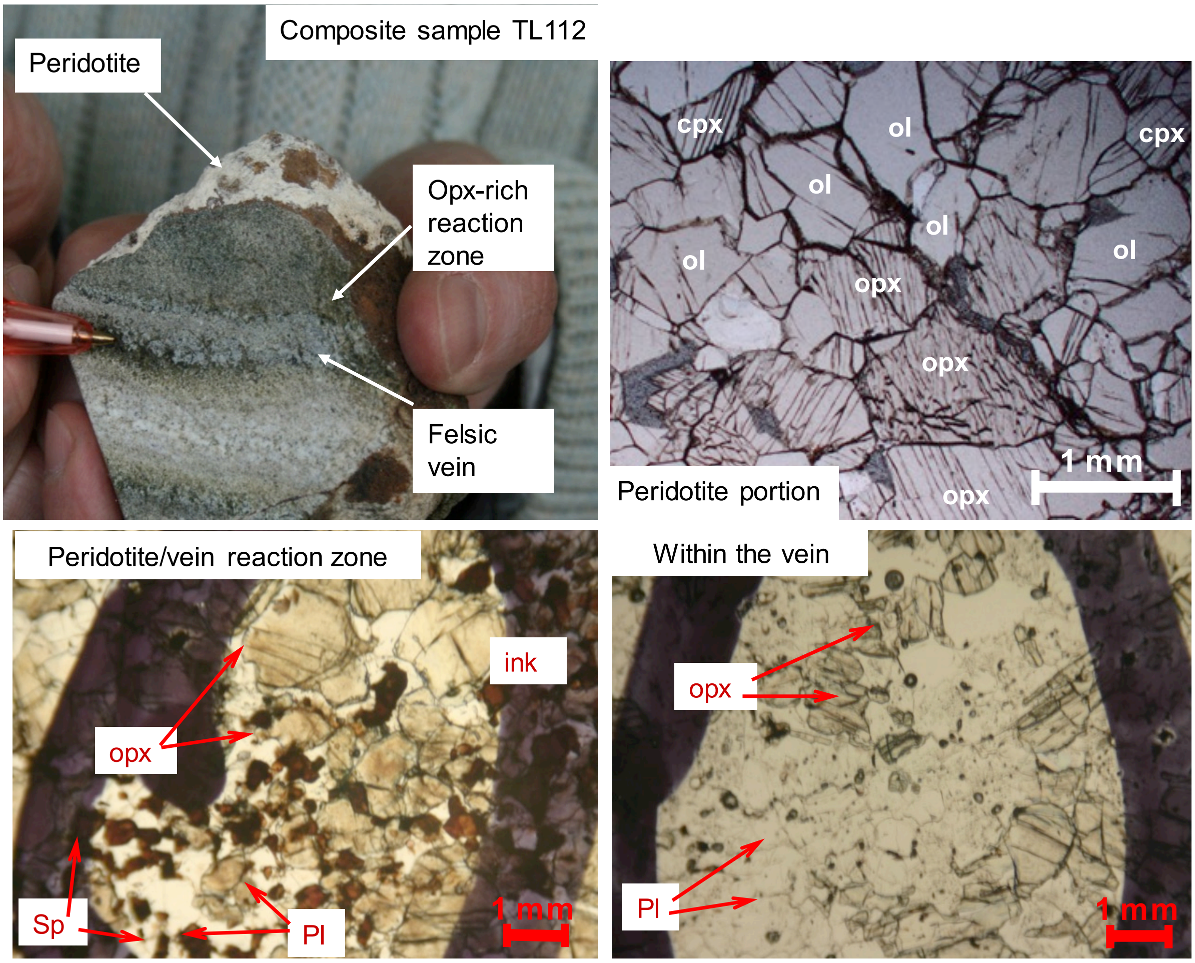

Supplementary Fig. 6. Hand specimen and microphotographs (at plane polarized transmitted light) of composite xenolith TL112.

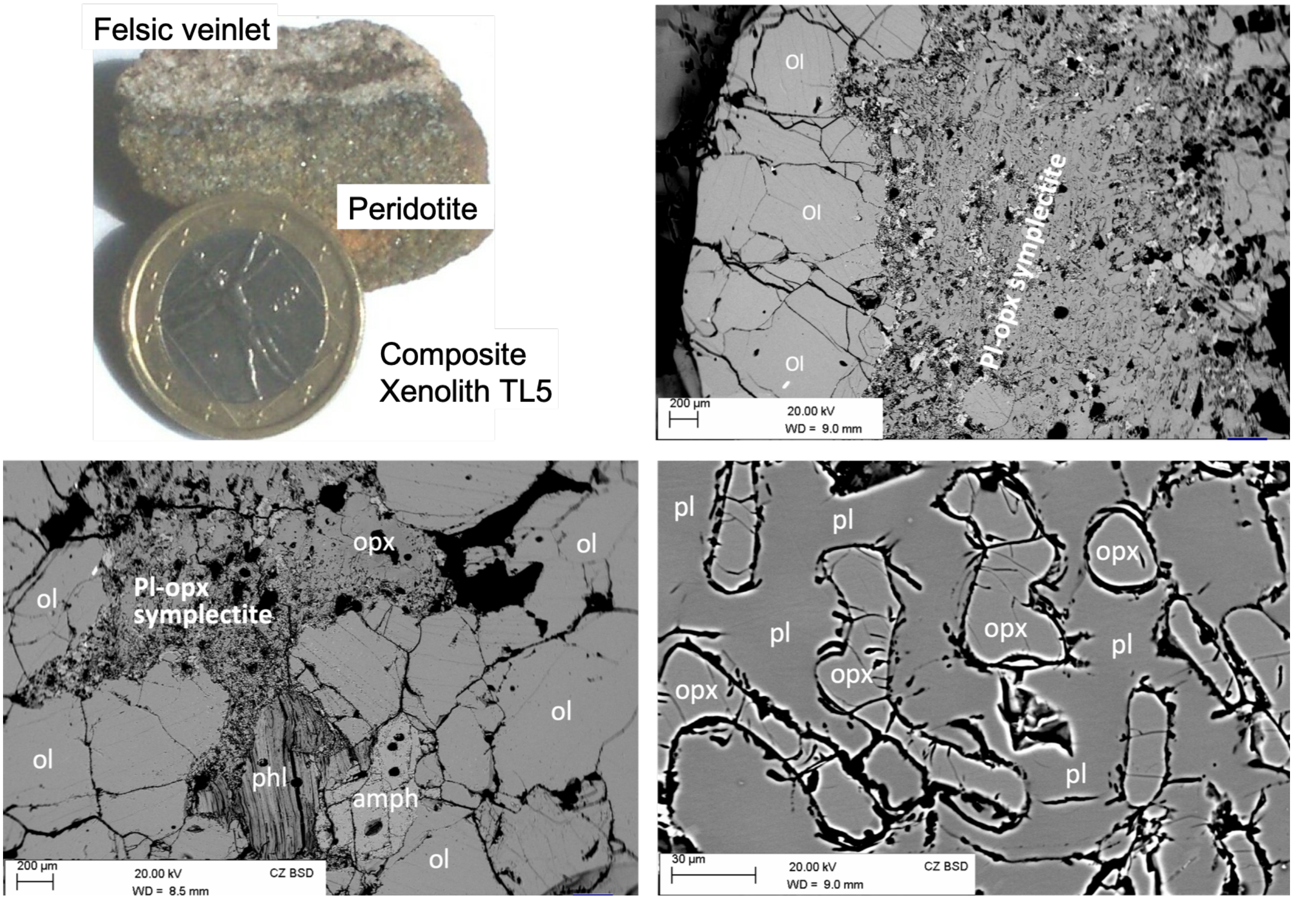

Supplementary Fig. 7. Hand specimen and SEM backscattered images of composite xenolith TL5. In this case the felsic vein is mainly made by a fine grained symplectite of plagioclase and orthopyroxene, containing well developed crystals of amphibole and phlogopite.

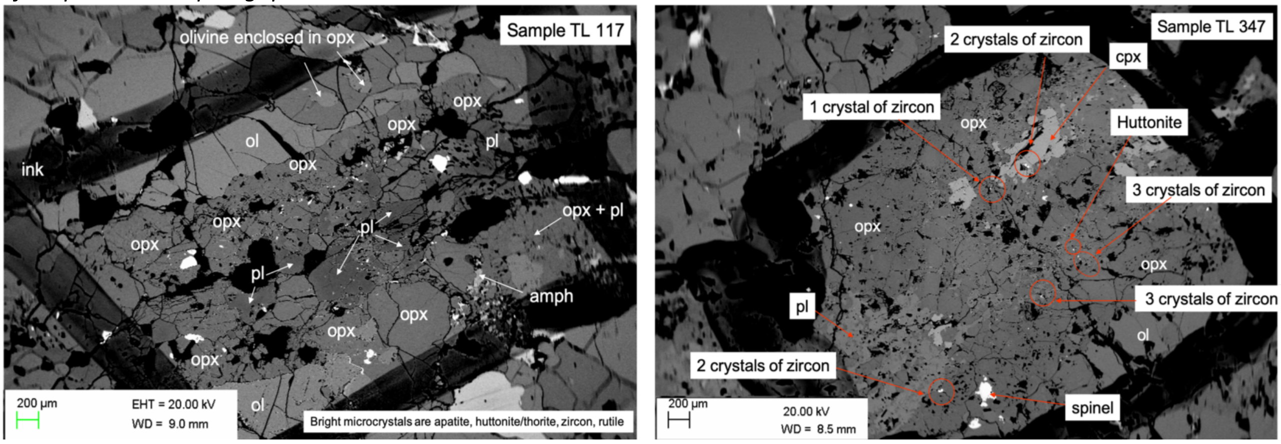

Supplementary Fig. 8. SEM backscattered images of millimetric felsic veinlets in composite xenoliths TL117 and TL347, where amphibole, apatite, thorite/huttonite, zircon, rutile have been observed.

2.3. Other xenoliths from Tallante

Other xenoliths found in the Tallante volcanic field have norite composition, being entirely composed by plagioclase and orthopyroxene and could represent felsic veins of thicker size with respect to those observed in composite xenoliths. Furthermore, other xenoliths having a metasedimentary origin have been recognised and studied by Vielzeuf (1983) and Bianchini et al.

(2013; 2015). They are characterised by silica/alumina-rich bulk compositions and display the following paragenesis: quartz, plagioclase, garnet, spinel, ilmenite ± orthopyroxene ± sillimanite ± graphite. These metasedimentary xenoliths equilibrated at 0.7 ± 0.1 GPa and 1050 ± 100 °C, i.e. at P–T conditions overlapping those recorded by the peridotite parageneses. Geobarometry therefore suggests the presence of a transitional MOHO, i.e. of an intimate association (an interlayering) of crust and mantle lithologies characterising the crust–mantle boundary (CMB; Bianchini et al., 2013; 2015).

3. Comparison between the lithospheric mantle section of Tallante and those from the neighbouring ultramafic bodies of Ronda and Beni Bousera.

Geological and petrological hypotheses proposed for the lithospheric mantle section of Tallante have to be coherent with what observed in the neighbouring ultramafic bodies of Ronda and Beni Bousera. Accordingly, the intimate association of crust and mantle rocks inferred by the study of Tallante xenoliths conforms to the field evidence provided by the neighbouring massifs of Ronda and Beni Bousera, where the exhumed fossil Crust Mantle Boundary (CMB) is characterised by mylonites and melanges (Thompson Lundeen, 1978; Van der Wal and Vissers, 1996; Tubía, eta al. 2004; Morishita et al. 2009; Platt et al., 2013). These mylonitic domains could reflect deep trans-lithospheric shear zones (Afiri et al. 2011; Vauchez et al., 2012) that favour inter-fingering/juxtaposition of distinct crustal and mantle lithologies. In these interlayered CMB boundaries, partial melting preferentially involved crustal domains typically characterised by lower solidus conditions. In our view, the resulting crustal melts, usually characterised by silica-oversaturation, escaped and migrated from the source region, veined the surrounding peridotite domains and also induced an orthopyroxene-rich metasomatic aureole.

In this scenario, felsic dykes of Miocene age crosscutting the peridotite bodies of Ronda and Beni Bousera (Rossetti et al., 2010 and references therein) could represent hectometre scale analogues of the little vein that we observe in the studied mantle xenoliths from Tallante. Coherently, according to Pereira et al. (2003) granitic dykes of Ronda have high $\delta^{18}O$ (up to 13.6 ‰) and seem to influence also the surrounding ultramafic rocks ($\delta^{18}O$ up to 10.0 ‰).

4. Analytical methods

4.1. Oxygen isotopic analyses

Oxygen isotope data were measured at the Consiglio Nazionale delle Ricerche — Istituto di Geoscienze e Georisorse (CNR — Institute for Geosciences and Earth Resource) of PISA by laser

fluorination (Sharp, 1990), reacting with 1 to 1.5 mg of olivine, orthopyroxene, clinopyroxene, plagioclase and spinel crystals in F_2 gas atmosphere. We employed a 25 W CO2 laser operating at a wavelength of 10.6 µm to irradiate the samples, and pure fluorine desorbed at 290 °C from hexafluoropotassium-nickelate salt as a reagent (Asprey, 1976). Three pre-fluorination steps were made before measuring new sets of analyses, in order to remove the moisture in the sample holder and the line. The O_2 produced during laser fluorination together with excess fluorine was passed through potassium chloride salt and excess fluorine was converted into a potassium-fluoride salt and chlorine gas. A cryogenic trap cooled at liquid nitrogen temperature was used to freeze chlorine.

After purification, O_2 was trapped over a cold finger filled with 5A zeolites (Sharp, 1995), and then transferred to a Finnigan Delta Plus Mass Spectrometer for oxygen isotope analysis. In-house laboratory standard Quartz Merck Standard (QMS) was measured at the beginning of each analytical session; after the standard samples reached the accepted values, mineral samples sequence started. 5 to 6 standards were measured during each set of analyses. The average $\delta^{18}O$ value of QMS is 14.05 ± 0.17‰ (1σ) and the $\delta^{18}O$ value of NBS30 is 5.24 ± 0.15‰ (1σ). All $\delta^{18}O$ values are relative to SMOW. At least two fragments were analysed for each mineral, and the variation within the same sample is less than the precision of standards. The results of oxygen isotope analyses are reported in supplementary table 1.

4.1. Strontium and neodymium isotopic analyses

Sr and Nd isotopic compositions were also determined at the CNR-IGG of Pisa on handpicked minerals leached in hot 6.2 M HCl for 45 minutes and rinsed several times in ultraclean water. Measurements were obtained by a Finnigan MAT 262 V multi-collector mass-spectrometer following separation of Sr and Nd using conventional ion-exchange procedures. Measured $^{87}Sr/^{86}Sr$ ratios were normalized to $^{86}Sr/^{88}Sr$ = 0.1194, $^{143}Nd/^{144}Nd$ ratios to $^{146}Nd/^{144}Nd$ = 0.7219. During the collection of isotopic data, replicate analyses of the Sr SRM-NIST 987 ($SrCO_3$) isotopic standard gave an average $^{86}Sr/^{88}Sr$ value of 0.710253 ± 13 (2σ, N = 30), whereas the Nd isotopic standard JNdi-1 (Tanaka et al., 2000) gave an average $^{143}Nd/^{144}Nd$ value of 0.512098 ± 8 (2σ, N = 25). The Sr and Nd Isotope data are reported in supplementary table 2.

5. *Relationships between oxygen isotope composition and mineral mode in unveined mantle xenoliths*

The following supplementary Figures 9a and 9b report $\delta^{18}O_{opx}$ *vs.* orthopyroxene/clinopyroxene and orthopyroxene/olivine modal values, respectively. They serve to emphasize that there is a relationship between modal proportion (and specifically orthopyroxene enrichment) and the oxygen isotope composition far from the felsic veins.

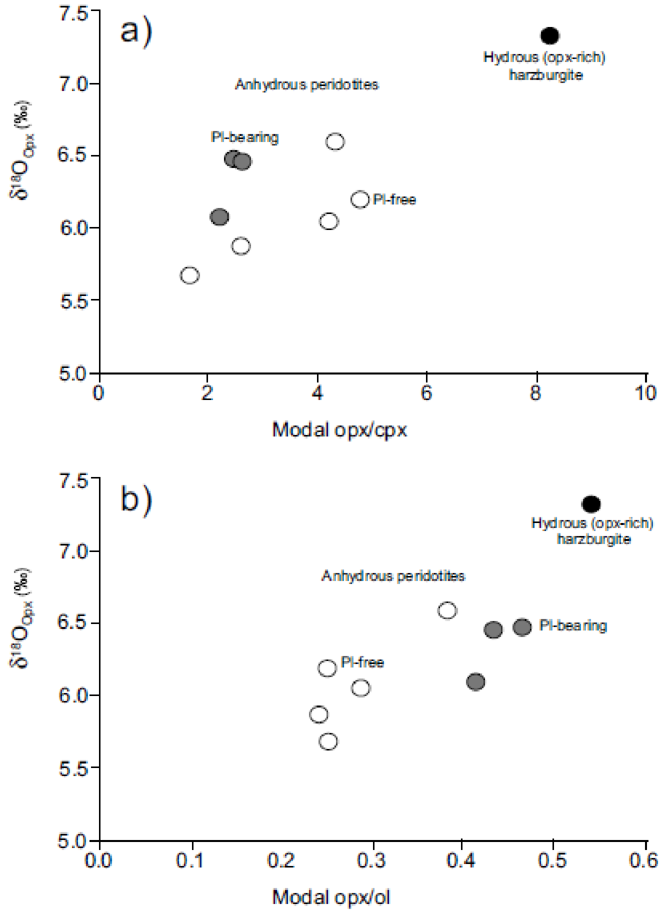

Supplementary Fig. 9. Relationships between modal proportions (Beccaluva et al., 2004) and $\delta^{18}O_{opx}$.

6. Rationale for oxygen diffusion modelling

The veins are approximated as a bimineralic (opx+pl) system, and the phase with lower diffusion rates (opx) is chosen to calculate how long a crustal signature can survive at mantle conditions. The measurements made on mineral separates preclude to exactly place the analysed orthopyroxene within the vein and/or within the reaction zone. We therefore refer to a "generic" orthopyroxene crystallised from the injected melt (ideally at the edge of the vein), having high $\delta^{18}O$ value, and could be in at the contact with "any" lherzolite and peridotite domain bearing

average mantle composition. Mantle lherzolite is modally dominant and supposed not to change its $\delta^{18}O$ value. The equation of Crank (1975) for a semi-infinite medium adjacent to a plane of constant composition is used, assuming constant P, T, f_{H2O} conditions. The diffusion-based calculations were performed using the parameters reported in supplementary table 3. The blue line ribbon represents the results obtained using the oxygen self-diffusion coefficient along a and c lenghts by Ingrin et al. (2001).

References

Afiri, A., Gueydan, F., Pitra, P., Essaifi, A. & Précigout, J. Oligo-Miocene exhumation of the Beni-Bousera peridotite through a lithosphere-scale extensional shear zone. *Geodin. Acta* **2**4, 49-60 (2011).

Arai, S., Shimizu, Y. & Gervilla, F. Quartz diorite veins in a peridotite xenolith from Tallante, Spain: implications for reactions and survival of slab-derived SiO2-oversaturated melts in the upper mantle. *Proceedings of the Japan Academy, Series B* **79**, 145–150 (2003).

Ancochea, E. & Nixon, P.H. Xenoliths in the Iberian Peninsula. In: Nixon, P.H. (Ed.), Mantle Xenoliths. John Wiley, pp. 119–124 (1987).

Beccaluva, L., Bianchini, G., Bonadiman, C., Siena, F. & Vaccaro, C. Coexisting anorogenic and subduction-related metasomatism in mantle xenoliths from the Betic Cordillera (southern Spain). *Lithos* **75**, 67–87 (2004).

Benito, R., Lòpez Ruiz, J., Cebrià, J.M., Hertogen, J., Doblas, M., Oyarzun, R. & Demaiffe, D., Sr and O isotope constraints on source and crustal contamination in the high-K calc-alkaline and shoshonitic Neogene volcanic rocks of SE Spain. *Lithos* **46**, 773–802 (1999).

Bianchini, G., Beccaluva, L., Bonadiman, C., Nowell, G., Pearson, G., Siena, F. & Wilson M., Evidence of diverse depletion and metasomatic events in harzburgite–lherzolite mantle xenoliths from the Iberian plate (Olot, NE Spain): Implications for lithosphere accretionary processes. *Lithos*, **94**, 25-45 (2007).

Bianchini, G., Beccaluva, L., Bonadiman, C., Nowell, G.M., Pearson, D.G., Siena, F. & Wilson M., Mantle metasomatism by melts of HIMU piclogite components: new insights from Fe-lherzolite xenoliths (Calatrava Volcanic District, Central Spain). *London Geological Society, Special Publication* **337**, 107-124 (2010).

Bianchini, G., Beccaluva, L., Nowell, G.M., Pearson, D.G. & Siena, F. Mantle xenoliths from Tallante (Betic Cordillera): insights into the multi-stage evolution of the south Iberian lithosphere. *Lithos* **124**, 308-318 (2011).

Bianchini, G., Braga, R. & Langone A. Crustal xenoliths from Tallante (Betic Cordillera, Spain): insights into the crust-mantle boundary. *Geol. Mag.* **150**, 952 – 958 (2013).

Bianchini, G., Braga, R., Langone, A., Natali, C., Tiepolo M. Metasedimentary and igneous xenoliths from Tallante (Betic Cordillera, Spain): Inferences on crust–mantle interactions and clues for post-collisional volcanism magma sources. *Lithos* **220-223**, 191-199 (2015).

Boudreau, A. E. PELE—a version of the MELTS software program for the PC platform. *Comput. and Geosci.* **25**, 201-203 (1999)

Capedri, S., Venturelli, G., Salvioli-Mariani, E., Crawford, A.J. & Barbieri, M. Upper mantle xenoliths and megacrysts in alkali basalts from Tallante, South-Eastern Spain. *Eur. J. Miner.* **1**, 685–699 (1989).

Cebriá, J.M., López-Ruiz, J., Carmona, J. & Doblas, M. Quantitative petrogenetic constraints on the Pliocene alkali basaltic volcanism of the SE Spain Volcanic Province. *J. Volcanol. Geotherm. Res.* **185**, 172–180 (2009).

Coltorti, M., Bonadiman, C., Faccini, B., Grégoire, M., O'Reilly, S.Y. & Powell, W. Amphiboles from suprasubduction and intraplate lithospheric mantle. *Lithos* **99**, 68–84 (2007).

Conticelli, S., Guarnieri, L., Farinelli, A., Mattei, M., Avanzinelli, R., Bianchini, G., Boari, E., Tommasini, S., Tiepolo, M., Prelević, D. & Venturelli, G. Trace elements and Sr–Nd–Pb isotopes of K-rich, shoshonitic, and calc-alkaline magmatism of the Western Mediterranean Region: genesis of ultrapotassic to calc-alkaline magmatic associations in a post-collisional geodynamic setting. *Lithos* **107**, 68–92 (2009).

Crank J. The mathematics of diffusion. Clarendon, Oxford p. 414 (1975)

Duggen, S., Hoernle, K., van den Bogaard, P. & Garbe-Schönberg, D. Post-collisional transition from subduction- to intraplate type magmatism in the westernmost Mediterranean: evidence for continental-edge delamination of subcontinental lithosphere. *J. Petrol*. **46**, 1155–1201 (2005).

Duggen, S., Hoernle, K., Klügel, A., Geldmacher, J., Thirlwall, M., Hauff, F., Lowry, D. & Oates, N. Geochemical zonation of the Miocene Alborán Basin volcanism (westernmost Mediterranean): geodynamic implications. *Contrib. Mineral. Petrol.* **156**, 577–593 (2008).

Dupuy, C., Dostal, J. & Boivin, P.A. Geochemistry of ultramafic xenoliths and their host alkali basalts from Tallante, southern Spain. *Mineral. Mag*. **50**, 231–239 (1986).

Faccenna, C., Piromallo, C., Crespo-Blanc, A., Jolivet, L., & Rossetti, F. Lateral slab deformation and the origin of the western Mediterranean arcs. *Tectonics* **23**, TC1012 (2004).

Hidas, K., Konc, Z., Garrido, C.J., Tommasi, A., Vauchez, A., Padrón-Navarta, J. A., Marchesi, C., Booth-Rea, G., Acosta-Vigil, A., Szabó, C., Varas-Reus, M.I. & Gervilla, F. Flow in the western Mediterranean shallow mantle: Insights from xenoliths in Pliocene alkali basalts from SE Iberia (eastern Betics, Spain). *Tectonics* **35**, 2657–2676 (2016).

Ingrin, J., Pacaud, L., Jaoul, O. Anisotropy of oxygen diffusion in diopside. Earth Planet. Sci. Lett. 192, 347–361 (2001).

Kogarko, L.N., Ryabchikov, I.D., Brey, G.P., Fernández Santin, S. & Pacheco, H. Mantle rocks uplifted to crustal levels: diffusion profiles in minerals of spinel-plagioclase lherzolites from Tallante, Spain. *Geochem. Int*. **39**, 355–371 (2001).

Konc, Z., Marchesi, C., Garrido, C.J., González-Ji-ménez, J.M., Griffi n, W.L., Alard, O., Hidas, K., O'Reilly, S.Y., & Pearson, N.J., Provenance and evolution of the western Mediterranean lithospheric mantle beneath the eastern Betics (S. Spain): insights from in-situ analyses of Os isotopes and platinum-group elements in sulphides from the Tallante mantle xenoliths.Geophysical Research Abstracts, v. 15, EGU12929 (2012).

Marchesi, C., Konc, Z., Garrido C.J., Boschc, D., Hidas, K., Varas-Reus, M.I. & Acosta-Vigil, A. Multi-stage evolution of the lithospheric mantle beneath the westernmost Mediterranean: Geochemical constraints from peridotite xenoliths in the eastern Betic Cordillera (SE Spain). *Lithos* **276**, 75-89.

Martelli M., Bianchini G., Beccaluva L. & Rizzo A. Helium and argon isotopic compositions of mantle xenoliths from Tallante and Calatrava, Spain. *J. Volcanol. Geotherm. Res.* **200**, 18-26 (2011).

Mattei, M., Riggs, N.R., Giordano, G., Guarnieri, L., Cifelli, F., Soriano, C.C., Jicha, B., Jasim, A., Marchionni, S., Franciosi, L. and Tommasini, S., 2014. Geochronology, geochemistry and geodynamics of the Cabo de Gata volcanic zone, Southeastern Spain. Italian Journal of Geosciences, 133(3), pp.341-361.

Morishita, T., Arai, S., Ishida, Y., Tamura, A. Constraints on the evolutionary history of aluminous mafic rocks in the Ronda peridotite massif (Spain) from trace-element compositions of clinopyroxene and garnet. *Geochem. J.* **43**, 191-206 (2009).

Platt, J.P., Behr, W.M., Johanesen, K. & Williams, J.R. The Betic-Rif Arc and its orogenic hinterland: A review. *Annu. Rev. Earth Planet. Sci*. **41**, 14.1–14.45 (2013).

Pereira, M.D., Shaw, D.M. & Acosta, A. Mobile trace elements and fluid-dominated processes in the Ronda peridotite, southern Spain. *Canad. Mineral*. **41**, 617-625.

Puga, E., Fanning, M., Díaz de Federico, A., Nieto, J.M., Beccaluva, L., Bianchini, G. & Díaz Puga M.A. Petrology, geochemistry and U-Pb geochronology of the Betic Ophiolites: Inferences for Pangaea break-up and birth of the westernmost Tethys Ocean. *Lithos* **124**, 255-272 (2011).

Rampone, E., Vissers, R.L.M., Poggio, M., Scambelluri, M. & Zanetti, A. Melt migration and intrusion during exhumation of the alboran lithosphere: the Tallante mantle xenolith record (Betic Cordillera, SE Spain). *J. Petrol.* **51**, 295–325 (2010).

Rossetti, F., Theye, T., Lucci, F., Bouybaouène, M.L., Dini, A., Gerdes, A., Phillips, D. & Cozzupoli, D. Timing and modes of granite magmatism in the core of the Alboran Domain (rif chain, northern Morocco): implications for the Alpine evolution of the western Mediterranean: Tectonics, v. 29, TC2017 (2010).

Shimizu, Y., Arai, S., Morishita, T., Yurimoto, H. & Gervilla, F. Petrochemical characteristics of felsic veins in mantle xenoliths from Tallante (SE Spain): an insight into activity of silicic melt within the mantle wedge. *Trans. Roy. Soc. Edinb., Earth Sci*. **95**, 265–276 (2004).

Sharp, Z.D., A laser-based microanalytical method for the in situ determination of oxygen isotope ratios in silicates and oxides. *Geochim. Cosmochim. Acta*, **54**, 1353-1357 (1990).

Shimizu, Y., Arai, S., Morishita, T. & Ishida, Y. Origin and significance of spinel–pyroxene symplectite in lherzolite xenoliths from Tallante, SE Spain. *Mineral. Petro*l. **94**, 27–43 (2008).

Tanaka, T., Togashi. S., Kamioka, H. & Amakawa, H. JNdi-1 A neodymium isotopic reference in consistency with LaJolla neodymium. *Chem. Geol.* **168**, 279–281 (2000).

Thompson Lundeen, M. Emplacement of the Ronda peridotite, Sierra Bermeja, Spain. GSA Bulletin 89, 172–180 (1978).

Tubía, J.M., Cuevas J. & Esteban, J.J. Tectonic evidence in the Ronda peridotites, Spain, for mantle diapirism related to delamination. *Geology* **32**, 941–944 (2004).

Turner, S.P., Platt, J.P., George, R.M.M., Kelley, S.P., Pearson, D.G. & Nowell, G.M. Magmatism associated with orogenic collapse of the Betic–Alboran domain, SE Spain. *J. Petrol.* **40**, 1011–1036 (1999).

Van der Wal, D. & Vissers, R.L.M. Structural petrology of the Ronda peridotite, SW Spain: deformation history. J. Petrol. 37, 23-43 (1996).

Vauchez, A., Tommasi, A. & Mainprice, D. Faults (shear zones) in the Earth's mantle. *Tectonophysics* **558–559**, 1-27 (2012).

Vielzeuf, D., 1983. The spinel and quartz associations in high grade xenoliths from Tallante (S.E. Spain) and their potential use in geothermometry and barometry. *Contrib. Mineral. Petrol.* **82**, 301–311.

Wortel, M.J.R. & Spakman, W. Subduction and slab detachment in the Mediterranean-Carpathian region, *Science* **290**, 1910-1917.

Supplementary Table 1. Oxygen isotopic composition of minerals from Tallante mantle xenoliths

**Unveined xenoliths**	**sample**	**mineral**	**$\delta^{18}O$ (‰)**	**std. err. mean**
Anhydrous peridotite xenoliths				
Hz	TL1	ol	5.74	0.12
		opx	6.05	0.11
		cpx	5.03	0.08
Lh	TL14	ol	5.19	0.08
		opx	6.45	0.04
		cpx	6.52	0.14
		sp	5.11	-
		pl	6.99	-
Lh (cpx-poor)	TL16	ol	5.78	0.20
		opx	5.88	0.12
		cpx	5.38	-
Lh	TL20	ol	5.49	0.13
		opx	6.47	0.08
		cpx	5.93	0.18
		pl	6.57	0.12
Lh (cpx-poor)	TL 22	ol	5.76	-
		opx	6.59	-
		cpx	6.09	-
Hz	TL24	ol	5.48	0.02
		opx	6.19	-
		cpx	5.61	5.61
Lh	TL45	ol	5.52	-
		opx	6.08	-
		cpx	6.03	-
		sp	4.35	-
		pl	6.32	-
Lh	TL53	ol	5.65	0.11
		opx	5.68	0.13
		cpx	5.46	0.04
		sp	4.47	0.06
Hydrous opx-rich xenoliths				
Amph-bearing Hz	TL 23	ol	6.99	
		opx	7.32	0.01
		cpx	7.06	0.09

**Composite xenoliths with felsic veins**	**sample**	**mineral**	**$\delta^{18}O$ (‰)**	**std. err. mean**
Felsic vein (3 cm thick)	TL112	pl	10.56	0.15
	felsic vein	opx	9.84	0.05
	border	opx	8.28	0.14
Host matrix	TL112	ol	5.73	0.13
	peridotite	opx	6.47	-
		cpx	6.21	0.03
		sp	4.20	0.10
Felsic vein (0,8 cm thick)	TL5	pl	9.63	0.10
		opx	8.02	0.17
Felsic veinlet (1 mm thick)	TL117	pl	7.33	0.19
Host matrix	TL117	cpx	6.13	0.05
Felsic veinlet (1 mm thick)	TL347	pl	7.58	0.05
Host matrix	TL347	cpx	5.96	0.18

Legend:

Standard error of the mean are reported for duplicated analyses.

Abbreviations: Hz= harzburgite, Lh=lherzolite, ol=olivine, opx=orthopyroxene, cpx=clinopyroxene, pl=plagioclase, sp=spinel.

Supplementary Table 2. Sr and Nd radiogenic isotopes carried out on mineral separates.

**Unveined xenoliths**	**sample**	**mineral**	**$^{87}Sr/^{86}Sr$**	**2δ**	**$^{143}Nd/^{144}Nd$**	**2δ**	**note**
Anhydrous peridotite xenoliths							
Hz	TL1	cpx	0.70368		0.51280		*
Lh	TL14	cpx	0.70213		0.51335		*
Lh (cpx-poor)	TL16	cpx	0.70413		0.51284		*
Lh	TL20	cpx	0.70298		0.51312		*
Lh (cpx-poor)	TL 22	cpx	0.70302		0.51305		*
Hz	TL24	cpx	0.70476		0.51248		*
Lh	TL45	cpx	0.70272		0.51313		*
Lh	TL53	cpx	0.70312		0.51311		*
Hydrous opx-rich xenoliths							
Hz	TL 23	cpx	0.70692		0.51213		*
**Composite xenoliths with felsic veins**							
Felsic vein (3 cm thick)	TL112	pl	0.71266	±1	0.51260	±2	**
surrounding peridotite	TL112	cpx	0.70588	±1	0.51260	±1	**
Felsic vein (0.8 cm thick)	TL5	Pl			*0.512452*		*
millimetric vein	TL117	pl	0.70409	±1	0.51282	±2	**
surrounding peridotite	TL117	cpx	0.70380	±1	0.51292	±2	**
millimetric vein	TL347	pl	0.70289	±1	0.51303	±3	**
surrounding peridotite	TL347	cpx	0.70286	±1	0.51318	±2	**

Legend:

* Data from: Beccaluva et al. (2004; Lithos 75, 67–87); Bianchini et al. (2011; Lithos 124, 308–318)

** New original data carried out by TIMS at the IGG-CNR of Pisa

Abbreviations: Hz= harzburgite, Lh=lherzolite, cpx=clinopyroxene; pl=plagioclase.

Supplementary Table 3. Assumptions and parameters adopted in the diffusion model

$\delta^{18}O_{ol} = 5.59$

$\delta^{18}O_{opx} = 6.12$

$\delta^{18}O_{cpx} = 6.10$

$\delta^{18}O_{opx\_vein} = 9.84$

$\delta^{18}O_{opx\_vein/matrix} = 8.28$

$\delta^{18}O_{pl\_vein} = 10.56$

$\delta^{18}O_{pl} = 6.96$

Radius cristalli μm

250

$$\frac{\delta^{18}O_t - \delta^{18}O_m}{\delta^{18}O_i - \delta^{18}O_m} = \mathrm{erf}\,\frac{X}{2\sqrt{Dt}}$$

Crank's (1975) diffusion equation for a semi-infinite medium adjacent to a plane of constant composition

Experimentally determined diffusion coefficients for oxygen in diopside

E (kJ/mol)<br>O diffusion in diopside	E<br>(kJ/mol)	D_0<br>(m2/s)	D (m2/s)<br>at 1,100°C
Legend:			
Connolly and Muehlenbachs (1988) [2]	404	6.30E-04	2.69E-19
Farver (1989) parallel c [3]	226	1.50E-10	3.79E-19
Farver (1989) perpendicular c [3]	226	2.80E-12	7.07E-21
Elphick and Graham (1990) [4]	351	9.00E-07	3.99E-20
Ryerson and McKeegan (1994) [5]	457	4.30E-04	1.77E-21
Ingrin et al. (2001) a,c [6]	259	1.00E-10	1.40E-20
Ingrin et al. (2001) b [6]	323	1.58E-09	8.16E-22

1. Crank (1975) The mathematics of diffusion. Clarendon, Oxford p. 414
2. Connolly, C., Muehlenbachs, K., 1988. Contrasting oxygen diffusion in nepheline, diopside and other silicates and their relevance to isotopic systematics in meteorites. Geochim. Cosmochim. Acta 52, 1585–1591.
3. Farver, J.R., 2010. Oxygen and hydrogen diffusion in minerals. In: Zhang, Y., Cherniak, D.J. (Eds.), Diffusion inMinerals and Melts. Reviews in Mineralogy and Geochemistry, 72. Mineral. Soc. Am, Chelsea, pp. 447–507.
4. Elphick S.C. and Graham C.M., 1990. Hydrothermal oxygen diffusion in diopside at 1 kb, 900- 1200 C, a comparison with oxygen diffusion in forsterite, and constraints on oxygen isotope disequilibrium in peridot&e nodules. Terra abstracts 2, 72 (abstr.).
5. Ryerson, F.J., McKeegan, K.D., 1994. Determination of oxygen self diffusion in akermanite, anorthite, diopside, and spinel: implications for oxygen isotopic anomalies and the thermal histories of Ca–Al-rich inclusions. Geochim. Cosmochim. Acta 58, 3713–3734.
6. Ingrin, J., Pacaud, L., Jaoul, O., 2001. Anisotropy of oxygen diffusion in diopside. Earth Planet. Sci. Lett. 192, 347–361.
